# Ultrasound-guided ethyl alcohol injection to the deep branch of the ulnar nerve to relieve hand spasticity in stroke patients: A case series

**DOI:** 10.1515/tnsci-2020-0188

**Published:** 2021-10-01

**Authors:** Min Cheol Chang, Gyu-Sik Choi, Mathieu Boudier-Revéret

**Affiliations:** Department of Physical Medicine and Rehabilitation, College of Medicine, Yeungnam University, Namku, Taegu, Republic of Korea; Cheokbareun Rehabilitation Clinic, Pohang-Si, Gyeonsangbuk-Do, South Korea; Department of Physical Medicine and Rehabilitation, Hôtel-Dieu du Centre Hospitalier de l’Université de Montréal, 3840, Saint-Urbain St., Montreal, QC, H2W 1T8, Canada

**Keywords:** ulnar nerve, muscle spasticity, stroke

## Abstract

Hand spasticity with a flexor pattern is a common problem affecting stroke patients and can result in pain, contractures, esthetic concerns, skin maceration, and overall loss of function. Poststroke (≥6 months) hemiparetic adult patients having a Modified Ashworth Scale (MAS) score of ≥1 for metacarpophalangeal flexion and thumb adduction spasticity were selected to receive an ultrasound-guided 20% ethyl alcohol block performed perineurally at the level of the deep branch of the ulnar nerve. Their MAS scores were evaluated pretreatment at 1 month and the change in MAS scores was assessed using Wilcoxon’s test. The threshold for statistical significance was set at *p* < 0.05. The mean MAS score for the flexor muscles of the 5 MCP joints and for thumb adduction was reduced from 3.3 ± 0.5 at pretreatment to 0.9 ± 0.5 at 1 month after the injection for the 10 patients. One month after the injection, the MAS scores were significantly reduced compared with those at pretreatment (*p* < 0.001), without complications. These are encouraging results showing that ultrasound-guided alcohol blocks of the deep branch of the ulnar nerve are safe and can help chronic stroke patients with metacarpophalangeal flexion and thumb adduction spasticity at 1 month.

Limb spasticity is a common problem in patients with stroke. Given that spasticity can directly affect motor function due to muscle tightness and joint stiffness in the affected limb, its management is an important issue in the field of stroke rehabilitation [1]. In particular, the hand is one of the body parts in which spasticity most frequently occurs. With hand function, humans manipulate objects and connect with their environment; therefore, impaired hand function due to spasticity decreases stroke patients’ ability to perform activities of daily living and deteriorates their quality of life [1,2]. In addition, when the degree of spasticity is severe, limited hand motion can result in hygiene problems with macerated and foul-smelling skin in the palm and also contractures [2]. Therefore, proper management of hand spasticity is important.

Spasticity of the hand typically presents with a flexor pattern and interferes with hand function by limiting joint extension. Spasticity of the intrinsic muscles of the hand limits the extension of the metacarpophalangeal (MCP), the flexion of interphalangeal joints, and the abduction of the thumb [3,4]. To control hand spasticity, many therapeutic methods such as oral medication, stretching exercises, orthoses, and botulinum toxin injection can be used. However, the effects of stretching and oral medication are often limited, while botulinum toxin injections are expensive. Moreover, the treatment for hand spasticity requires the injection of botulinum toxin into several muscles of the hand which can be time-consuming and painful. In 1987, Keenan et al. reported successful treatment of intrinsic spasticity in the hands of stroke patients with phenol neurolysis of the deep branch of the ulnar nerve (DBUN), which is responsible for motor innervation of most of the intrinsic muscles of the hand, employing an open motor branch block in Guyon’s canal by exposing surgically the DBUN and injecting it subepineurally with a 5% solution of phenol in glycerine [5]. However, this method necessitated extensive resources to be performed in an operating room. Since the motor and sensory branches of the ulnar nerve are in proximity at the level of the wrist, mostly the sensory superficial branch (it also supplies the palmaris brevis muscle) can be easily damaged in the process of neurolysis with phenol or alcohol if improper guidance is used.

To prevent damage to the superficial branch of the ulnar nerve, we performed ultrasound-guided alcohol neurolysis of the DBUN to manage flexor spasticity in the hands of stroke patients.

## 1 Description of case series

We prospectively recruited ten stroke patients among the patients who visited the university hospital or the local rehabilitation clinic for a routine follow-up after discharge after inpatient rehabilitation treatment. Hemiparetic stroke patients were recruited into this study according to the following inclusion criteria: (1) ≥6 months after stroke onset, (2) finger flexor and thumb adduction spasticity (a Modified Ashworth Scale [MAS] score of ≥1), (3) age ≥20 years old, (4) no history of peripheral nerve injury, and (5) no history of any invasive procedure (injection of botulinum toxin, alcohol, or phenol) for the treatment of spasticity for at least 6 months before the initiation of this study. We did not change any drugs or perform any procedures that might have affected the spasticity during the study period. Ethyl alcohol injection and follow-up after the injection were performed in the university hospital for all the included patients.

**Informed consent:** Informed consent has been obtained from all individuals included in this study.**Ethical approval:** The research related to human use has been complied with all the relevant national regulations, institutional policies, and in accordance with the tenets of the Helsinki Declaration and has been approved by the Yeungnam university hospital review board.

## 2 Ethyl alcohol injection

**Figure 1:**
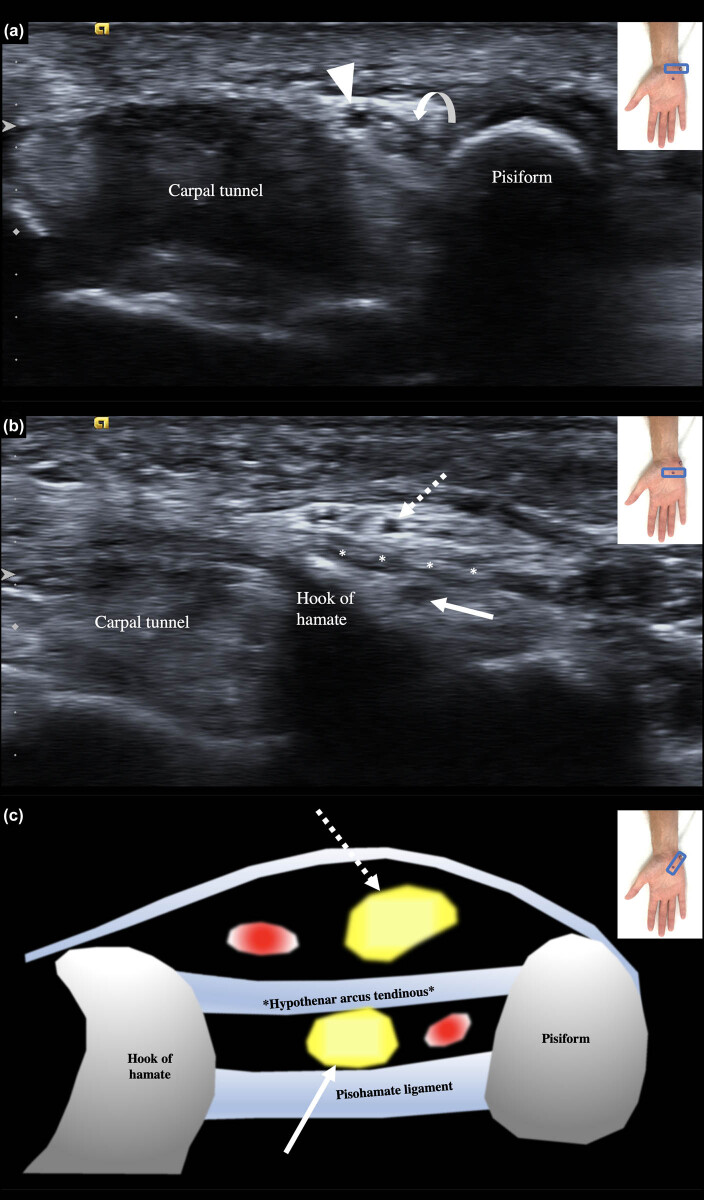
Grayscale ultrasound images showing the ulnar nerve in transverse axis at the level of the entry of Guyon’s canal, at the level of the pisiform bone, (a) and at the exit, at the level of the hook of hamate (b), where the injection was performed with an ulnar to radial in-plane approach. In (a), the ulnar nerve (curved arrow) is seen medial to the ulnar artery (arrowhead). In (b), the ulnar nerve has split in its superficial (dotted arrow) and deep (full arrow) branches, separated by the hypothenar arcus tendinous (asterisks). A schematic drawing (c) in the longitudinal axis of the pisohamate ligament of the superficial (dotted arrow) and deep (full arrow) branches of the ulnar nerve, accompanied respectively by the superficial palmar arch of the ulnar artery and the deep palmar branch of the ulnar artery (depicted as red dots), is presented.

Ethyl alcohol was injected around the DBUN on the affected side at the distal end of Guyon’s canal under US guidance (13–18 MHz linear probe, Acuson S2000, Siemens), with the patient in the supine position and the affected hand in a supinated position. All injections were performed by the same physiatrist with 10 years of experience in US-guided procedures. For the procedure, the US probe was placed at the level of the hook of the hamate, where the superficial and deep branches are separated by the hypothenar arcus tendinous and the deep branch courses above the pisohamate ligament (Figure 1). After identifying the deep and superficial branches of the ulnar nerve, 1.5 mL of 20% ethyl alcohol was injected perineurally near the DBUN with a 23 G, 1.5-inch needle, in a transverse view of the DBUN with an in-plane ulnar to radial approach.

## 3 Clinical evaluation

We used MAS scores to assess the degree of spasticity in the flexor muscles of the five MCP joints and of thumb adductors: 0, no increase in muscle tone; 1, slight increase in muscle tone manifested by a catch and release or by minimal resistance at the end of the range of motion (ROM) when the affected part(s) was(were) moved in flexion or extension; 1+, slight increase in muscle tone manifested by a catch, followed by minimal resistance throughout the remainder (less than half) of the ROM; 2, a more marked increase in muscle tone through most of the ROM, but affected part(s) easily moved; 3, considerable increase in muscle tone, passive movement difficult; 4, affected part(s) rigid in flexion or extension. Categories 1+ to 4 were designated as 2–5 for the purpose of analysis. The degree of spasticity of the flexor muscles of the five MCP joints and of thumb adduction was assessed at pretreatment and 1 month after the injection in each patient.

## 4 Statistical analysis

Statistical analysis was conducted using SPSS version 22.0 (IBM Corp., Armonk, NY, USA). The change in MAS scores after alcohol injection was assessed using Wilcoxon signed rank test. The threshold for statistical significance was set at *p* < 0.05.

## 5 Results

Ten hemiparetic stroke patients (mean age = 59.5 ± 6.9 years, age range: 48–68 years, male:female = 6:4, infarct:hemorrhage = 6:4, mean period after the stroke onset = 20.4 ± 14.0 months, see Table 1 for complete details). None of the study participants were lost to follow-up, and no adverse effects, such as bleeding, dysesthesias, and infection, were observed. The mean MAS score for the flexor muscles of the 5 MCP joints and for thumb adduction was reduced from 3.3 ± 0.5 at pretreatment to 0.9 ± 0.5 at 1 month after the injection. At 1 month after the injection, the MAS scores were significantly reduced compared with those at pretreatment (*p* < 0.001).

**Table 1:** Details of the included patients and modified Ashworth scale scores pre- and post-treatment for metacarpophalangeal flexion and thumb adduction

	Age/sex	Stroke site	Period after the stroke onset (months)	MAS score at pretreatment	MAS score at 1 month after treatment
Patient 1	56/F	Right hemiplegia due to left corona radiata infarct	14	2	0
Patient 2	66/F	Right hemiplegia due to left corona radiata infarct	10	2	1
Patient 3	52/M	Left hemiplegia due to left basal ganglia intracerebral hemorrhage	18	2	0
Patient 4	68/M	Right hemiplegia due to left thalamic intracerebral hemorrhage	24	3	1
Patient 5	59/F	Left hemiplegia due to right basal ganglia intracerebral hemorrhage	60	3	1+
Patient 6	64/M	Left hemiplegia due to right posterior limb infarct	17	2	1
Patient 7	53/M	Left hemiplegia due to right corona radiata infarct	12	2	1
Patient 8	64/F	Right hemiplegia due to left fronto-temporo-parietal lobe infarct	9	2	1
Patient 9	65/M	Left hemiplegia due to pons infarct	24	2	1
Patient 10	48/M	Left hemiplegia due to right basal ganglia intracerebral hemorrhage	20	3	1

## 6 Discussion

In this study, we performed neurolysis of DBUN with 20% ethyl alcohol in ten patients with stroke, and the MCP flexion as well as the thumb adduction spasticity of their hands was significantly reduced at 1 month.

Neurolysis with ethyl alcohol is known to be an effective method of controlling spasticity after stroke. Alcohol contributes to the reduction of spasticity by denaturing proteins, resulting in the splitting of myelin sheaths [6]. Neurolysis has been performed with success on various nerves, such as the musculocutaneous and obturator nerves, the motor branch of the tibial nerve, and the motor nerves innervating the finger flexors [7,8,9,10]. During these procedures, the targeted nerve is usually identified using a nerve stimulator. However, in the case of neurolysis of the DBUN, due to the anatomical proximity of sensory branches, collateral damage could occur, causing neuropathic pain. Therefore, to date, there has been a reluctance to perform ulnar nerve neurolysis in clinical practice. At the wrist, the ulnar nerve innervates most of the intrinsic muscles of the hand, including the deep head of flexor pollicis brevis, palmar and dorsal interossei, third and fourth lumbricals, adductor pollicis, as well as hypothenar (abductor digiti minimi, opponens digiti minimi, flexor digiti minimi brevis) muscles [11]. In our study, by conducting the neurolysis of the DBUN with a low volume of ethyl alcohol under US guidance, we were able to perform an accurate perineural injection selectively affecting the motor branch of the ulnar nerve. In addition, the effect of chemical neurolysis with alcohol usually lasts 3–6 months; therefore, if necessary, repeating the injection can be performed with spasticity recurrence [12].

Limitations of this exploratory case series include a small number of patients, a short follow-up period, absence of a control group and of blinding, evaluation of the MAS as the only outcome, time after stroke ranging from 9 to 60 months, and the absence of formal pre-alcohol injection evaluation of joint contractures with a DBUN local anesthetic block, which has been suggested before performing a surgical neurectomy [5,13]. Future studies with a higher number of poststroke patients, studying both more acute and chronic stroke patients, preselected for the absence of joint contractures, randomized to blinded control or treatment groups, with more pre- and posttreatment outcomes including functional, spasticity, and pain scales, would be highly pertinent.

In conclusion, we successfully conducted US-guided neurolysis of the motor branch of the ulnar nerve in Guyon’s canal using 20% ethyl alcohol. Owing to the ability of US to visualize even small nerves, we could avoid perineural injection of the sensory branch of the ulnar nerve during the procedure. Our study showed that alcohol neurolysis of the DBUN is a useful and safe therapeutic method for flexor spasticity in the hands of stroke patients.
